# Health habits and other characteristics of dietary supplement users: a review

**DOI:** 10.1186/1475-2891-13-14

**Published:** 2014-02-06

**Authors:** Annette Dickinson, Douglas MacKay

**Affiliations:** 1 President, Dickinson Consulting LLC, 3432 Denmark Avenue, #350, St. Paul, MN 55123, USA; 2 V.P., Scientific and Regulatory Affairs, Council for Responsible Nutrition, 1828 L Street, N.W., Suite 510, Washington, D.C 20036, USA

**Keywords:** Dietary supplements, Healthy habits, Surveys of supplement use

## Abstract

Dietary supplements are used by half to two-thirds of American adults, and the evidence suggests that this usage is one component of a larger effort to develop a healthier lifestyle. Dietary supplement users tend on average to be better educated and to have somewhat higher incomes than nonusers, and these factors may contribute to their health-consciousness. Dietary supplement use also tends to be more prevalent among women than among men, and the prevalence of use increases with age in both men and women. Numerous surveys document that users of dietary supplements are significantly more likely than nonusers to have somewhat better dietary patterns, exercise regularly, maintain a healthy weight, and avoid tobacco products. While supplement users tend to have better diets than nonusers, the differences are relatively small, their diets have some substantial nutrient shortfalls, and their supplement use has been shown to improve the adequacy of nutrient intakes. Overall, the evidence suggests that users of dietary supplements are seeking wellness and are consciously adopting a variety of lifestyle habits that they consider to contribute to healthy living.

## Introduction

Numerous surveys indicate that somewhere between half and three-quarters of American adults use dietary supplements – mostly vitamins and minerals, but also omega-3 fatty acids, other bioactives such as lutein, and other food components such as fiber [1-4]. Together, these surveys provide meaningful insight into not only the demographics but also the health and lifestyle habits of dietary supplement users. In many published articles, however, the health and lifestyle habits are noted in passing but not emphasized and therefore possibly not fully appreciated within the nutrition, public health, and medical communities. The purpose of this review is to accumulate and compare the available data on the health habits of dietary supplement users, to provide a sharper insight into the generally positive lifestyle choices of this large segment of the American population.

The evidence from numerous surveys shows that dietary supplement users are more likely than nonusers to adopt a number of positive health-related habits [1-4]. These include better dietary patterns, exercising regularly, maintaining a healthy body weight, and avoidance of tobacco products. The differences between supplement users and nonusers seen in various studies are not huge but are consistently observed, indicating that supplement users are making a greater effort to seek health and wellness.

Dietary supplement users differ from nonusers in demographic characteristics as well. For example, supplement use in adults has been consistently reported to increase with age, income, and education; and within each age group, women are more likely to use supplements than men [1-4]. These demographic factors may also be related to observed differences in health awareness and health habits of supplement users, compared to nonusers – but the differences in health habits consistently remain significant even after adjusting for demographic factors, in most studies and for most population groups.

### What is the “real” prevalence of dietary supplement use?

The prevalence of dietary supplement use reported in a survey depends in part on the exact question posed in the survey. In the National Health and Nutrition Examination Surveys (NHANES), people are asked whether they used dietary supplements in the month prior to the interview. A month is a very short time, so this question will not capture all the supplement use that may have occurred seasonally or occasionally over the past year. It will capture people who use supplements on a regular basis, plus those occasional users who happen to have taken the supplement in the month before the interview. Based on this question, the NHANES surveys that collected data in 1999–2000, 2003–2006, and 2007–2010 have reported that about half of adult Americans use dietary supplements (range of 49% to 54%) [1-3]. This may be an underestimate of overall use, since it fails to include all seasonal or occasional users. A series of nationally representative consumer surveys conducted by Ipsos Public Affairs for the Council for Responsible Nutrition (CRN) illustrates this point. The CRN surveys for the five-year period from 2007 to 2011 found that 48 to 53% (average of 50%) of consumers considered themselves “regular” users of dietary supplements, while the overall prevalence of supplement use was 64 to 69% (average of 66%) when occasional and seasonal users were included [5]. Results of these surveys suggest that the prevalence of *regular* supplement use among U.S. adults is about 50%, but the *overall* prevalence of supplement use may be closer to two-thirds of the adult population.

### Usage higher in women; increases with age

Many surveys confirm that dietary supplement use is more prevalent in older age groups than in younger adults, and in each age group usage is higher in women than in men [1-4]. Diet and health surveys suggest that women may be more health conscious than men; however the increased rates of supplement usage among women could also partially be attributed to the increased use of supplemental calcium and vitamin D among women for the purpose of maintaining bone health throughout the lifespan and preventing the onset of osteoporosis during aging. Table 1 shows the prevalence of supplement use in men and women in various age groups in the National Health and Nutrition Examination Surveys (NHANES) conducted in 2003–2006 [1]. As age increases, the prevalence of dietary supplement use in men increases from 36% to 66%, and the prevalence of use in women increases from 43% to 75%. In each age group, usage is 7 to 14% higher in women than in men (on average, 10% higher).

**Table 1:** **Prevalence of dietary supplements use in men and women, by age group, NHANES 2003–2006**[1]

	**Men**	**Women**
Age 19-30	36%	43%
Age 31-50	44%	55%
Age 51-70	58%	72%
Age >70	66%	75%

In the Multiethnic Cohort study, researchers found that the prevalence of dietary supplement use in more than 100,000 healthy adults over the age of 45 was 58% in men and 72% in women, which are the same levels reported in NHANES 2003–2006 for men and women ages 51–70 [4]. The Vitamins and Lifestyle (VITAL) survey, which sampled more than 45,000 adults 50 or more years of age, reported that over 75% of respondents used at least one of the 17 dietary supplements examined [6].

### Race, ethnicity

The prevalence of supplement use varies with race or ethnicity and is generally found to be highest in non-Hispanic Whites (hereafter simply Whites). In the NHANES surveys in 1999–2000 and 2003–2006, dietary supplement use was almost 60% in Whites, 36% in Blacks, and about 34% in Hispanics [1,2]. These differences were not observed or were less marked in the Multiethnic cohort, where supplement use was reported by 69% of Whites, 68% of Japanese Americans, 66% of African Americans, 62% of Latinos, and 53% of native Hawaiians [4].

### Number of supplements taken

Many users of dietary supplements take more than one product, but taking only one remains the most common finding. In NHANES 1999–2000, 47% of supplement users took only one supplement, 23% took two, 13% took three, and 17% took four or more [2]. In NHANES 2003–2006, more than half of supplement users reported taking only one, but 10% took more than 5 [1].

### Prevalence of multivitamin use among supplement users

Most users of dietary supplements take a multivitamin (with or without minerals), whether or not they also take other products. In NHANES 1999–2000, among supplement users, 67% took a multivitamin [2]. In NHANES 2003–2006, 74% of supplement users took a multivitamin [1]. The term “multivitamin” is not clearly defined, for purposes of scientific study, and surveys have applied various definitions. In the NHANES surveys, a “multivitamin” has generally been defined as a product containing three or more vitamins, with or without minerals, but this can vary from one NHANES study to another [1,2]. The term “multivitamin” in commercial use typically denotes a product containing all or most of the vitamins, whereas a product containing numerous minerals in addition to the vitamins is typically labeled as a “multivitamin/multimineral”.

In the Multiethnic cohort, most supplement users took a multivitamin, and vitamin C was the most commonly used supplement, after multivitamins [4]. In CSFII 1994–96, among supplement users over age 50, 70% of women and 74% of men who took supplements took a multivitamin [7]. In a consumer survey sponsored by the Council for Responsible Nutrition in 2011, multivitamins were the most commonly used supplement, reported by 71% of supplement users [5].

### Frequency and duration of supplement use

Users of dietary supplements typically take their chosen products every day, and many stick with their supplement regimen for years. Thus, supplement use does not appear to be a passing fancy, but more of a planned strategy that is often maintained over the long haul. In NHANES 2003–2006, most supplement users (79%) reported taking the supplements every day within the past 30 days [1]. In NHANES 1999–2000, 85% of those who took a multivitamin took it daily, as did 82% of those who took vitamin C and 90% of those who took vitamin E [2]. Also, 25% of those who took a multivitamin had been taking it for 5 years or more, as had 38% of those who took vitamin C and 34% of those who took vitamin E [2]. Many respondents had used the supplements for 10 years or more, including 14% of those who took a multivitamin, 29% of those who took vitamin C, and 22% of those who took vitamin E [2].

xIn the Multiethnic healthy cohort, over 60% of white or Japanese-American supplement users, about 50% of black or native Hawaiian supplement users, and about 40% of Latino supplement users reported taking multivitamins regularly for 5 years or more [4]. Longterm use of vitamin A, vitamin C, vitamin E, calcium and iron was also substantial.

### Prevalence of supplement use in U.S. has increased over time

Dietary supplement use among American adults is not a new phenomenon, but is documented throughout the NHANES series of surveys, beginning over 40 years ago, in 1971 [1-3,8-10]. As shown in Table 2, the prevalence of dietary supplement use in adults was already substantial in 1971 and increased steadily in every NHANES survey until the late 1990s. The prevalence was 23% in the early 1970s, 35% in the late 1970s, 42% in 1988–94, and then rose to about 50% by 1999 and remained fairly steady around that level through 2010 [1-3,8-10]. Although it is sometimes assumed that there was a sharp increase in supplement use after the passage of the Dietary Supplement Health and Education Act (DSHEA) in 1994, the data do not show a unique jump in usage at that time. The increase from one NHANES survey to the next was 12% between the first and second and 7% between the second and third. An increase of 10% was reported from the 1988–1994 survey to the 1999–2000 survey, spanning the period from six years before to six years after passage of DSHEA. Since 1999–2000, the prevalence of supplement use reported in NHANES has remained roughly steady at about half the adult population (49 to 54%) [1-3].

**Table 2:** Increase in prevalence of dietary supplement use over time as shown in a series of NHANES surveys

**Survey**	**Prevalence of dietary supplement use**
NHANES I, 1971-74	23% of adults [10]
NHANES II, 1976-80	35% of adults [9]
NHANES III, 1988-1994	42% of adults [8]
NHANES 1999-2000	52% of adults [2]
NHANES 2003-2006	54% of adults [1]
NHANES 2007-2010	49% of adults [3]

### Higher education is associated with supplement use

Surveys consistently show that supplement usage is higher in people with more education than in people with less. In NHANES 2003–2006, supplements were used by 61% of those with more than a high school education and by only 37% of those with less than a high school education [1].

In NHANES 1999–2000, supplements were used by 62% of those with more than a high school education, 48% of those with a high school education, and only 35% of those with less than a high school education [2]. In the Multiethnic cohort and the VITAL study, educational level was also positively associated with supplement use [4,6].

### Obese people are less likely to be supplement users

One healthy habit that appears to be adopted by supplement users is to make more of an effort to maintain a normal body weight, or at least to avoid obesity. In NHANES 2003–2006, supplement use was reported by 56% of people with normal weight and by 57% of overweight people, as compared to only 48% of obese subjects [1]. Similar findings were reported in NHANES 1999–2000 [2]. In the Multiethnic cohort, obese persons were also found to be less likely to adopt supplement use [4].

### People who exercise are more likely to use supplements

Another healthy habit that tends to be somewhat more common among supplement users than nonusers is the practice of getting regular physical exercise. In NHANES 1999–2000, supplement use was 59% in subjects who reported moderate or vigorous physical activity, compared to 43% in subjects who reported no physical activity [2]. In the Multiethnic cohort, engaging in regular physical activity was also positively associated with supplement use [4].

### Smoking

Avoiding smoking is universally accepted as a desirable health or lifestyle practice, and users of dietary supplements tend to be people who have never smoked or who have given up smoking. Current smokers are less likely to be supplement users. In NHANES 1999–2000, supplement use was reported by 61% of former smokers and 52% of people who had never smoked, compared to 43% of current smokers [2]. In the Multiethnic cohort, current smokers were also found to be less likely to report supplement use [4].

### Beer, wine and spirits

In the first NHANES survey (1971–74), it was observed that drinking wine was associated with a higher prevalence of dietary supplement use, while there was no association with beer drinking [10]. It was speculated that this was related to the somewhat higher socioeconomic status of supplement users.

In NHANES 1999–2000, supplement use was highest (72%) in people who drink wine more than 4 times a month, compared to 59% in subjects who drink wine 1–4 times a month and 47% in those who do not drink wine, while there was little or no relationship to beer drinking [2]. Supplement use was also higher in those who drink distilled spirits more than 4 times a month (62%) than in those who do not drink distilled spirits (51%). As in NHANES I, these relationships are likely related to socioeconomic status. Drinking wine or spirits more than four times a month in no way suggests excessive intake.

### Reasons for using dietary supplements

Surveys typically inquire about dietary supplement use but not about the reasons for using such products. A report on NHANES 2007–2010 for the first time reports national survey data on consumers’ reasons for using dietary supplements [3]. The Council for Responsible Nutrition (CRN) has conducted two series of surveys, one series on consumer use of supplements and one series on health professionals’ use of supplements, and these surveys also include an analysis of the reasons given for supplement use [5,11-13]. Table 3 shows comparative data from these three sources, relating to the reasons people give for using dietary supplements. From the CRN surveys, the table shows the 2011 data on consumer reasons for using supplements and the 2009 data on dietitians’ reasons for using supplements [5,11]. Improving or maintaining overall health, supporting bone health, and filling nutrient gaps rank at or near the top in all these surveys.

**Table 3:** Percent of subjects citing various reasons for using dietary supplements, in NHANES 2007–2011 and in two CRN surveys, one in 2011 on consumer use of dietary supplements and one in 2009 on use by dietitians

**Reason for use**	**NHANES 2007–2010 [**[3]**]**	**CRN Consumer survey, 2011 [**[5]**]**	**CRN survey, dietitians, 2009 [**[11]**]**
Overall health:			
Improve overall health	45%		
Maintain health	33%		
Overall health/wellness		58%	53%
Bone health	25%	30%	58%
Supplement the diet, fill nutrient gaps	22%	42%	42%
Prevent health problems	20%	26%	
Heart health, lower cholesterol	15%	29%, 19%	25%, 16%
Boost immunity, prevent colds	15%	32%, 17%	25%, 21%
Healthy joints, prevent arthritis	12%	20%	15%
Enhanced energy	11%	31%	15%
Skin, hair and nails	5% (skin only)	17%	13%
Bowel or colon health, digestive health	5%	15%	26%
Eye health	4%	13%	9%
Mental health or focus, concentration	4%	13%	
Weight loss, weight management	3%	14%	6%

(Includes questions asked in at least two of the three surveys; response rounded to nearest percent).

### Are people with disease more likely to use supplements?

Some surveys have examined the relationship between supplement use and medical conditions. Analysis of the 2001 and 2003 California Health Interview Surveys identified 1576 cancer survivors and 4951 subjects with no history of cancer [14]. When supplement usage was examined, the authors found that “a diagnosis of cancer, by itself, does not have an independent effect on supplement use [14]”. However, most cancer survivors have other chronic conditions (cardiovascular, respiratory, diabetes, arthritis, etc.). The prevalence of using two or more dietary supplements was 52% in people with no noncancer chronic conditions, 64% in people with one noncancer chronic condition, and 71 to 76% in people with two or more noncancer chronic conditions [14].

In the Vitamins and Lifestyle (VITAL) survey, overall supplement use in more than 45,000 respondents was 75% [6]. Respondents were 50 to 75 years of age. While people with disease or health complaints were no more likely to be supplement users than people without disease, the number of supplements used was somewhat higher among people with a disease or health condition than among people without it. The conditions examined ranged from serious diseases such as heart disease and cancer to conditions such as arthritis and asthma to complaints such as chronic back pain, frequent heartburn, allergies, and chronic fatigue. About 55% of the subjects surveyed in VITAL had some health complaint and 45% had none. Those with some health complaint used an average of 1.5 supplements, while those without a health complaint used an average of 1.35 supplements [6]. About 22% of the respondents had some serious disease and 78% did not. Those with some serious disease used an average of 1.7 supplements, while those with no serious disease used an average of 1.6 supplements [6].

These studies do not suggest that people with disease suddenly become users of dietary supplements. Rather, it seems possible that people who are already supplement users may decide to add something extra to their regimen, relating to a health complaint or disease condition.

### Diet quality

People who use dietary supplements are more likely than nonusers to pay attention to their diets and to make an effort to improve dietary intake. In people over 50, in the CSFII 1994–96, the perceived importance of consuming a healthy diet was “a significant predictor of supplement use [7]”.

In the Multiethnic cohort, persons who consumed diets high in fat or low in fiber or low in fruit were less likely to use dietary supplements [4]. Thus, dietary supplement use was related to consuming better diets, in terms of being lower in fat and higher in fiber and higher in fruits. The authors of the report on the Multiethnic cohort concluded that the findings “suggest that a ‘health conscious’ attitude predominates among dietary supplement users [4]”.

### Nutrient intake from multivitamins

In the Multiethnic cohort, nutrient intake specifically from multivitamins was examined. Among 159,017 people who responded to a 1999–2001 survey on nutrient intake from supplements, about 50% reported use of multivitamin products (with or without minerals) [15]. Median daily nutrient intakes from multivitamin/multimineral supplements were well above the RDA or AI for: vitamin A, thiamin, riboflavin, niacin, pantothenic acid, vitamin B-6, folate, vitamin B-12, vitamin E, zinc, copper, manganese, molybdenum, and chromium. Median intakes were close to or below the RDA or AI for biotin, vitamin C, and vitamin D. Median intakes of calcium and selenium were much less than the RDA or AI. Ninetieth percentile intakes of nutrients from multivitamins were below the UL except for vitamin A, niacin, and folate.

### Nutrient intakes of supplement users vs nonusers

An analysis of CSFII, 1994–96, reported that supplement users over the age of 50 were less likely than nonusers to have inadequate nutrient intakes from food alone (as compared to the EAR) [7]. Regular supplement use “reduced the percentage of older adults with inadequate intakes by at least three-fourths for most nutrients [7]”. The authors commented: “Supplements had a positive influence on nutrient adequacy for men and women aged 51 years and older. Whereas dietary modifications to improve intake are paramount, the use of supplements by older adults appears beneficial to attain nutrient adequacy [7]”.

Murphy et al. examined nutrient intake from food and supplements in subjects in the Multiethnic Cohort who took multivitamins (with or without minerals or other components), compared to people who did not take any vitamin or mineral supplements [16]. Nutrient intake from food only was calculated for people who did not use any multivitamins or other supplements included in the survey (38,374 men and 31,341 women). Nutrient intake from food only and from food plus supplements was calculated for people who used a multivitamin for two years or more but did not take any of the specified single supplements (11,125 men and 9,931 women).

The probability of nutrient adequacy was calculated for 17 nutrients. The “prevalence of dietary nutrient adequacy based on food intake alone was similar for multivitamin supplement users and nonusers [16]”. The average prevalence of adequacy for all 17 nutrients in men was 74% for nonusers and 76% for multivitamin users. The average prevalence of adequacy for women was 72% for nonusers and 75% for multivitamin users. When nutrients from food and supplements were included, the prevalence of adequacy for people who used multivitamins was 84% for men and 83% for women.

The Multiethnic Cohort study found that some people consumed more than the UL (Upper Level of Tolerable Intake) for some nutrients from food alone (niacin, folate, vitamin A, iron, zinc, calcium) [16]. When food plus supplement intake was included, over 50% of men and over 40% of women who used multivitamins consumed more than the UL for niacin and for folate. Over 15% consumed more than the UL for vitamin A, and over 10% consumed more than the UL for iron. More than 10% of men and almost 10% of women who used multivitamins consumed more than the UL for zinc. The UL is defined as the highest level of daily intake that is likely to pose no risk of adverse effects for almost all healthy people. As intakes increase above the UL, the risk of adverse effects may increase. More research is needed regarding the significance of intakes above the UL, especially for nutrients such as niacin, folate, vitamin A, and zinc, where the UL is only two or three times greater than the RDA. (For comparison, the ULs for vitamin C and vitamin E are, respectively, more than 20 and more than 60 times the RDA). It would be desirable for consumers and healthcare professionals to be aware of these levels, and companies that market dietary supplements should put a priority on tailoring formulations to provide meaningful supplementation without exceeding ULs.

Fulgoni, Bailey and co-authors examined sources of nutrient intake in NHANES 2003–2006 [17]. They found that many Americans of all ages failed to consume the Estimated Average Requirement (EAR) for many nutrients, when only naturally-occurring nutrients in foods were considered. The authors make no comparisons of nutrient intakes to Recommended Dietary Allowances (RDA), but obviously people who were below the EAR were even further below the RDA, which is by definition higher. Nutrients added in enrichment and fortification decreased the prevalence of intakes below the EAR, and the use of dietary supplements further decreased shortfalls [17]. For example, more than half of the respondents fell short of the EARs for vitamin A and calcium, when only naturally-occurring nutrients were considered. When enrichment and fortification were taken into account, the prevalence of shortfalls fell below 50% for both nutrients. When supplementation was taken into account, the prevalence of shortfalls for these two nutrients fell further, but remained above 33%.

These same authors separately examined nutrient intakes from food in 8,860 adults surveyed in NHANES 2003–2006 and calculated nutrient intakes of dietary supplement users as compared to nonusers [18,19]. They found that people who used dietary supplements had somewhat higher intakes of most nutrients from food alone (not counting the nutrients in dietary supplements) than people who were not supplement users. The prevalence of intakes below the EAR was substantial for many nutrients, in people who were not supplement users, as shown in Table 4. The prevalence of intakes below the EAR fell rather dramatically for users of dietary supplements, when the nutrients contributed by the supplements were taken into account, as also shown in Table 4. However, for some nutrients such as vitamin D and calcium, the prevalence of intakes below the EAR remained substantial (25% and 20%, respectively), even including supplemental intakes [18,19].

**Table 4:** Percent of adults with nutrient intakes below the Estimated Average Requirement (EAR), for nonusers of dietary supplements as compared to users of dietary supplements (DS users), in NHANES 2003-2006

**Nutrient**	**% Below EAR, nonusers**	**% Below EAR, DS users**
Vitamin A	58	2
Vitamin C	48	3
Vitamin D	96	25
Vitamin E	96	5
Folate	14	1
Calcium	51	20
Iron	8	0.6
Zinc	13	0.3

Nutrient intakes for DS Users include nutrients contributed by dietary supplements [18,19].

Supplement use among respondents in NHANES 2003–2006 somewhat increased the chance of exceeding the level designated as the Upper Level of Tolerable Intake (UL) for some nutrients [18,19]. For example, 1 to 2% of adult supplement users in various age groups had vitamin C intakes that were above the UL, and 1 to 5% of adult supplement users in various age groups had vitamin A intakes that were above the UL. Results for most minerals were similar, but the prevalence of intakes above the UL was higher for some minerals. For example, in people 51 to 70 years of age, 14% of men and 6% of women who used supplements had iron intakes above the UL.

### Special populations: users of food stamps

A study of nutrient intakes in food stamp recipients in the 1994–96 CSFII found that supplement usage was lower in food stamp recipients than in non-recipients [20]. (The program historically known as “food stamps” is now called the Supplemental Nutrition Assistance Program or SNAP, but the older term was used in the article and thus is used here.) USDA does not permit the use of food stamps for the purchase of dietary supplements, but many food stamp users purchase these products with their own resources. Among food stamp recipients, supplements were used by 41% of women and 26% of men, while among people who did not receive food stamps, supplements were used by 57% of women and 43% of men [20]. Supplement users had higher vitamin and mineral densities from food than nonusers – roughly 10 to 20 percent higher. They also scored higher on two measures of diet quality. However, these results were not uniform for all ethnic, gender, and age groups [20].

### What about people who use numerous supplements for many years?

A unique survey by Block and co-authors examined the health status of 278 people who had used numerous dietary supplements for 20 years or more [21]. They used products made by a major multilevel marketing company and took an average of 18 different products every day. These health status of these “heavy users” was compared to 602 nonusers and 176 users of a multivitamin only, matched for age and race, drawn from NHANES surveys. The products used daily by more than 50% of the heavy users included a multivitamin/mineral, B complex, vitamin C, carotenoids, vitamin E, calcium with D, omega-3 fatty acids, flavonoids, lecithin, alfalfa, coenzyme 10 with resveratrol, glucosamine, and an herbal immune supplement. Among women, the majority also consumed gamma linolenic acid and a probiotic supplement [21]. Among men, the majority also consumed zinc, garlic, saw palmetto, and a soy protein supplement.

“After adjustment for age, gender, income, education and body mass index, greater degree of supplement use was associated with more favorable concentrations of serum homocysteine, C-reactive protein, high-density lipoprotein cholesterol, and triglycerides, as well as lower risk of prevalent elevated blood pressure and diabetes [21]”.

## Conclusions

Dietary supplement use is prevalent among U.S. adults and is often associated with the adoption of other healthy habits that are generally encouraged as part of a healthier lifestyle. These healthy habits include efforts to consume a better diet, and supplement users tend to have somewhat higher intakes of nutrients from foods than nonusers of supplements. Even so, the dietary intakes of supplement users often fall short of requirements or recommendations, and their supplements make an important contribution toward nutrient intakes. Other healthy habits adopted by supplement users include efforts to participate in physical exercise, avoidance of obesity, and avoidance of smoking. It is important to recognize these characteristics of dietary supplement users and give supplement users appropriate credit for attempting to improve their overall wellness profile. The evidence indicates that users of dietary supplements tend to incorporate these products into their lifestyles as part of a broader focus on healthy living.

## Abbreviations

CRN: Council for responsible nutrition; DS: Dietary supplement(s); DSHEA: Dietary supplement health and education act of 1994; EAR: Estimated average requirement; NHANES: National health and nutrition examination survey; RDA: Recommended dietary allowance; SNAP: Supplemental nutrition assistance program (formerly food stamps); UL: Upper level of tolerable intake; VITAL: Vitamins and lifestyle survey.

## Competing interests

AD is a former employee of and a current consultant to the Council for Responsible Nutrition (CRN), a trade association of the dietary supplement industry. DM is Vice President for Scientific and Regulatory Affairs for the Council for Responsible Nutrition. Consulting funds to support the preparation of this article were provided by the CRN Foundation, a nonprofit organization that provides consumers, researchers, and healthcare professionals with information about the responsible use of dietary supplements as part of a healthy lifestyle.

## Authors’ contributions

AD prepared the initial draft of the manuscript and participated in all discussions and revisions. DM provided substantive critical review and comments, and both authors approved the final version submitted for publication.
